# Examining the associations between difficulties in emotion regulation and symptomatic outcome measures among individuals with different mental disorders

**DOI:** 10.3389/fpsyg.2023.944457

**Published:** 2023-03-14

**Authors:** Libby Igra, Sharon Shilon, Yogev Kivity, Dana Atzil-Slonim, Adi Lavi-Rotenberg, Ilanit Hasson-Ohayon

**Affiliations:** Department of Psychology, Bar-Ilan University, Ramat Gan, Israel

**Keywords:** emotion regulation, distress, depression, schizophrenia, emotional disorders

## Abstract

**Background:**

Difficulties in emotion regulation (ER) abilities have been found to play a central role in different psychiatric disorders. However, researchers rarely compare ER across different diagnostic groups. In the current study, we examined ER and its relation to functional and symptomatic outcome among three distinct diagnostic groups: people with schizophrenia (SCZ), people with emotional disorders (EDs; i.e., depression and/or anxiety), and individuals without any psychiatric diagnosis (controls).

**Methods:**

Participants in this study comprised 108 adults who requested psychotherapy at a community clinic in the year 2015 and between 2017 and 2019. Clients were interviewed and filled out questionnaires measuring depression, distress, and difficulties in ER abilities.

**Results:**

Results showed that individuals with psychiatric diagnoses reported higher levels of difficulties in ER abilities than did controls. Moreover, there were very few differences in levels of ER difficulty between SCZ and EDs. Further, the associations between maladaptive ER and psychological outcomes were significant in each diagnostic group, and especially for SCZ.

**Conclusion:**

Our study indicates that difficulties in ER abilities partially have a transdiagnostic nature, and that these difficulties are associated with psychological outcomes among both clinical populations and controls. There were very few differences in levels of ER ability difficulties between SCZ and EDs, suggesting that the two groups share difficulties in relating and responding to emotional distress. The associations between difficulties in ER abilities and outcome were more robust and stronger among SCZ than the other groups, highlighting the potential contribution of targeting ER abilities in the treatment of schizophrenia.

## 1. Introduction

Emotion regulation (ER) has been defined as a set of processes that people use to maintain optimal homeostatic arousal in order to facilitate goal-oriented functioning (Gross, 2015). By using these processes, one can adapt one’s emotional experience and its magnitude according to the contextual requirements. Based on conceptualizations that emphasize the contextual-dependent nature of adaptive ER (Cole et al., 1994; Thompson, 1994), Gratz and Roemer (2004) suggested an integrative multidimensional assessment of individuals` typical ways of understanding, relating, and responding to emotions. They proposed six complementary yet distinctive dimensions of ER abilities that include awareness of emotional responses, clarity of emotional responses, acceptance of emotional responses, access to emotion regulation strategies perceived as effective, controlling impulses when experiencing negative emotions, and engaging in goal-directed behaviors when experiencing negative emotions.

Deficits in ER abilities are considered risk factors for the development and maintenance of multiple forms of psychopathology (Glenn and Klonsky, 2009; Gratz et al., 2015; Monell et al., 2018). In addition to research on ER abilities that focus on dimensions of adaptive vs. maladaptive ways of responding to emotional distress (Gratz and Roemer, 2004), there is increasing literature on the application of particular ER strategies to influence the experience or expression of emotions (Gross and John, 2003; Garnefski and Kraaij, 2006; Webb et al., 2012; Gross, 2015). Studies on ER strategies have found specific maladaptive strategies (e.g., rumination, avoidance, and suppression) and adaptive strategies (e.g., problem solving, acceptance, and reappraisal) that play a significant role across a range of psychopathologies (Aldao et al., 2010). Recent accounts of ER suggest that these approaches are complementary; hence, deficits in ER abilities and the use of particular ER strategies both contribute to how adaptively emotions are regulated (Gross, 2015; Tull and Aldao, 2015; Doré et al., 2016).

Research linking difficulties in ER abilities and maladaptive ER strategies with clinical disorders include mood disorders (Hallion et al., 2018; Gonçalves et al., 2019; Miola et al., 2022), anxiety disorders (Mennin et al., 2005; Salters-Pedneault et al., 2006; Kashdan and Breen, 2008; Cisler et al., 2010), psychotic disorders (Lincoln et al., 2015; Ludwig et al., 2019), personality disorders (Linehan, 1993; Lynch et al., 2007), posttraumatic stress disorder (Tull et al., 2020), eating disorders (Clyne and Blampied, 2004; Bydlowski et al., 2005), and substance-related disorders (Fox et al., 2007; Sher and Grekin, 2007; Weiss et al., 2022). The growing body of literature demonstrating that ER is a clinically-relevant construct highlights the importance of targeting ER difficulties within therapeutic interventions. Accordingly, several interventions that target ER abilities and strategies have been developed, such as emotion-focused therapy (Greenberg, 2004), dialectical behavioral therapy (Linehan, 1993), ER therapy (Mennin and Fresco, 2014; Mennin et al., 2018), acceptance therapy, and mindfulness-based therapy (Hayes et al., 2009).

Although there are theories that posit ER as a transdiagnostic mechanism underlying numerous psychiatric difficulties and maladaptive behaviors (Campbell-Sills and Barlow, 2007; Bloch et al., 2010; Gratz and Tull, 2010a,b), it is not clear whether there are specific patterns of ER difficulties that characterize distinct diagnostic groups. Research comparing difficulties in ER abilities and related clinical outcomes across distinct diagnostic groups is rare. Previous research on ER and different psychopathology manifestations is mainly based on comparing ER strategies, not ER abilities (for example, see Aldao et al., 2010; Sheppes et al., 2015). Of note, a meta-analysis comparing ER strategies across diagnostic groups showed different patterns of maladaptive strategies between disorders (Aldao et al., 2010). However, it did not assess specific dimensions of ER abilities and it did not include clients with schizophrenia spectrum disorders, limiting the ability to compare their patterns of ER with those of other diagnostic groups.

In recent decades evidence has accumulated to suggest that individuals diagnosed with schizophrenia (SCZ) have challenges in choosing adaptive ER strategies, leading to less effective ER processes (Perry et al., 2011; Ludwig et al., 2019; Lawlor et al., 2020; Opoka et al., 2021). A recent meta-analysis that assessed the use of cognitive ER strategies among SCZ found that maladaptive ER strategies are more frequently reported, and adaptive strategies are less frequently reported, compared to among non-patients controls (Ludwig et al., 2019). In addition, SCZ report greater difficulties in emotional clarity and emotional acceptance than do controls (Lawlor et al., 2020). Of note, ER maladaptive strategies have been linked to the formation and maintenance of positive psychiatric symptoms among SCZ (Ludwig et al., 2019; Kimhy et al., 2020).

Although research on ER difficulties among SCZ is growing rapidly, research on ER abilities in accordance with the Gratz and Roemer (2004) multidimensional model remains scarce. In a sample of inpatients with serious mental disorders, all of the dimensions of difficulties in ER were related to depression, anxiety, and somatization symptoms (Fowler et al., 2014). Another study that assessed difficulties in ER abilities in a subclinical sample found that impulse control difficulties were associated with persecutory ideation (Westermann and Lincoln, 2011). However, to the best of our knowledge, no systematic evaluation of different dimensions of difficulties in ER abilities and their association with clinical outcomes among SCZ has yet been conducted, nor a comparison of these patterns with patterns among individuals with emotional disorders and controls.

Previous research comparing SCZ with depressed individuals in emotional deficits has yielded mixed results: Some studies found less emotional expressiveness among SCZ than among depressed individuals (for example, Yecker et al., 1999), whereas other studies found higher levels of expressiveness among SCZ (Berenbaum and Oltmanns, 1992). Interestingly, in a study that compared SCZ with depressed individuals and controls, the two diagnostic groups exhibited more impairments in facial emotion expression than did controls, but they also differed in the type of impairments they exhibited: Depressed participants had fewer spontaneous expressions of other-than-happiness emotions, but overall they appeared more expressive (Trémeau et al., 2005). In addition, SCZ and depressed participants reported more negative emotions than did controls, but the difference between the level and type of negative emotional experiences of SCZ and depressed participants remains unclear (Trémeau, 2006). Studies on ER strategies have found that SCZ and depressed individuals differ from controls, but the two clinical groups have similar negative emotions and maladaptive ER strategies (Livingstone et al., 2009). Finally, Lincoln et al. (2015) found similar difficulties among participants with SCZ and depression in ER skills, with a tendency toward even more pronounced difficulties among SCZ. Based on the inconclusive findings regarding emotional deficits, ER strategies, and ER skills that are related to ER abilities (Trémeau, 2006), in the current study, we compared SCZ, participants with emotional disorders, and controls in their ER abilities and related outcomes.

Emotion regulation abilities seem to play a significant role both in depression and anxiety disorders (Cisler et al., 2010; Gonçalves et al., 2019). According to the unified protocol framework (Moses and Barlow, 2006), individuals diagnosed with depression and anxiety disorders can be grouped together as facing emotional disorders (EDs). The functional model of EDs suggests that both depression and anxiety share similar difficulties and usage of maladaptive ER abilities that contribute to the persistence of emotional symptoms. The core difficulty according to the model is negative reactions to strong emotions, leading to a reliance on ineffective strategies that backfire and exacerbate emotion (Campbell-Sills and Barlow, 2007). In support of the theoretical assumptions of this model, there is a high lifetime comorbidity rate between depression and anxiety disorders, which has been estimated to be as high as 75% (Kessler et al., 2005; Brown and Barlow, 2009; Wilamowska et al., 2010).

Our first aim was to compare difficulties in ER abilities among SCZ, EDs, and controls. Although difficulties in ER abilities seem to characterize different diagnostic populations (Gratz and Tull, 2010a,b; Gratz et al., 2015), studies have rarely compared ER impairments of different clinical groups. Based on a review of emotion deficits that show SCZ and EDs face more deficits in emotional abilities than do controls (Trémeau, 2006), we hypothesized that SCZ and EDs would show more difficulties in ER abilities than would controls. As the differences between SCZ and EDs in emotional expression, experience, and regulation remain unclear (Trémeau, 2006; Livingstone et al., 2009; Lincoln et al., 2015), we did not formulate a hypothesis vis a vis specific differences in their ER abilities, and thus this analysis was exploratory.

Our second aim was to explore the associations of difficulties in ER abilities with depressive and general symptoms. Based on previous research linking specific ER dimensions with symptomatic and functional outcomes (Fowler et al., 2014; Ryan et al., 2016; Hallion et al., 2018; Gonçalves et al., 2019), we hypothesized that there would be associations between ER abilities and depressive symptoms and general symptoms among the three groups. Our third aim was to compare the strength of associations between difficulties in ER abilities and outcomes across the groups. Given the lack of existing literature comparing groups in the above-mentioned associations, these investigations were exploratory.

In order to investigate our research aims, we compared difficulties in ER abilities among three distinct diagnostic groups: individuals diagnosed with schizophrenia (SCZ), individuals diagnosed with emotional disorders (EDs; i.e., depression and/or anxiety), and individuals without a clinical diagnosis (controls). Then, we calculated the correlations between each difficulty in ER ability with functional and symptomatic outcomes. Finally, we compared the sizes of the correlations between the three groups. This research is expected to contribute to the understanding of disorder-specific deficits and assist in tailoring interventions that target ER abilities most effectively.

## 2. Method

### 2.1. Participants

Participants comprised 108 adults (55 men) between the ages of 20 and 58 (*M =* 38.52, *SD* = 9.47). The sample for the current research consists of clients who requested psychotherapy at the university community clinic during the year 2015 and between 2017 and 2019. The current study’s data are baseline data collected before the participants’ engagement in psychotherapy. All participants were Hebrew speakers, and the data were collected using validated Hebrew versions of the measures listed. Exclusion criteria were intellectual disability, neurological disorders, substance use problems, acute psychosis, and risk for suicidal behavior, based on the intake interview. The first subsample included 36 SCZ who enrolled in a metacognitive reflection insight therapy trial (Igra et al., 2022). The second subsample included 36 other clients who signed up for psychotherapy in the clinic and were diagnosed with major depression, dysthymia, and/or anxiety disorders. All diagnoses were determined according to the Mini-International Neuropsychiatric Interview 4.5 (MINI 4.5; Sheehan et al., 1998), which is part of a baseline intake in the clinic. The last subsample also included 36 clients who signed up for psychotherapy in the clinic but did not meet the criteria for any diagnosis. All participants approached the clinic voluntarily for psychotherapy.

As age and gender have been found to influence difficulties in ER abilities and their impact on outcome (Gratz and Roemer, 2004; Orgeta, 2009; Bender et al., 2012), participants were matched in gender and age (±3 years). When more than one match was available from a subsample, we randomly assigned a matching participant from the available participants. Of note, the initial subsample of SCZ included 50 participants. However, we were unable to match 14 individuals; therefore, only 36 participants from that subsample were compared to the EDs and control subsamples. Participants from the SCZ subsample that were included in the current study did not differ from participants in the SCZ subsample that were not included due to the matching procedure in the level of difficulties in ER abilities and their general distress levels, but they had higher levels of BDI scores.

### 2.2. Procedure

Following the obtaining of ethical approval for the study and the signing of a consent form, and prior to their engagement in psychotherapy, clients were interviewed at the clinic and were asked to fill out questionnaires. With the SCZ subsample, the reported measures were administered on a computer in the clinic, whereas with the other subsamples, the administration of scales was done *via* participants’ electronic devices at a time and place of their choosing.

### 2.3. Measures

#### 2.3.1. Mini-international neuropsychiatric interview 4.5

A structured interview of Axis-I disorders of the DSM-IV-TR. The interviewers were interns in clinical or rehabilitation psychology (Sheehan et al., 1998).

#### 2.3.2. Beck depression inventory-II

A 21-item self-report measure of depression that asks respondents to rate the severity of their depressive symptoms during the previous 2 weeks using a variable Likert-type scale (i.e., 19 items use a four-point scale, and two items use a seven-point scale; Beck et al., 1996). Individual item scores are summed to create a total severity score ranging from 0 to 63. Cronbach’s α estimate of internal consistency in the current study for the depression score was 0.914.

#### 2.3.3. Outcome questionnaire-45

A 45-item self-report, five-point Likert-type scale questionnaire, is designed to measure clients’ changes in distress and functioning over the course of their mental health treatment, with higher scores representing higher symptom severity. It has been found to have adequate test–retest reliability (0.84) and a high internal consistency, Cronbach’s alpha = 0.93 (Kadera et al., 1996; Lambert et al., 1996). Cronbach’s α estimate of internal consistency in the current study was 0.926. In line with extensive studies, including the one on scale development (Lambert et al., 1996) that consistently showed high correlations between subscales of distress and functioning and total score, we used a total score in the current study.

#### 2.3.4. Difficulties in ER scale

A 36-item self-report measure that assesses individuals’ difficulties in ER (Gratz and Roemer, 2004). The DERS is based on a clinically multidimensional conceptualization of ER that was developed to be valid for a wide variety of psychological difficulties and relevant to clinical applications and treatment development (Gratz and Tull, 2010a,b). The DERS is composed of six dimensions reflecting potential difficulties: (a) lack of awareness of emotional responses, (b) lack of clarity of emotional responses, (c) non-acceptance of emotional responses, (d) limited access to ER strategies perceived as effective, (e) difficulties controlling impulses when experiencing negative emotions, and (f) difficulties engaging in goal-directed behaviors when experiencing negative emotions. The items are statements that are rated on a five-point Likert-type scale ranging from 1 (almost never) to 5 (almost always). After recoding the inverse items, individual item scores are summed to create a total score of difficulties in ER, or are summed for each subscale score. Higher scores indicate greater difficulties in ER. The questionnaire has been used extensively in research among clinical populations (Fowler et al., 2014; Rosenstein et al., 2018; Scoglio et al., 2018), and has good reliability and validity, including high overall internal consistency (α = 0.93) and Cronbach’s alpha >0.80 for each subscale (Gratz and Roemer, 2004). Cronbach’s α estimate of internal consistency for the DERS score in the current study was 0.93.

### 2.4. Analytic strategy

First, we used between-subjects ANOVA to examine differences between the diagnostic groups (1 = SCZ, 2 = EDs, 3 = controls) in the ER variables: (a) lack of awareness of emotional responses; (b) lack of clarity of emotional responses; (c) non-acceptance of emotional responses; (d) limited access to ER strategies perceived as effective; (e) difficulties controlling impulses when experiencing negative emotions; and (f) difficulties engaging in goal-directed behaviors when experiencing negative emotions. Second, we examined differences between the diagnostic groups in the outcome variables: (a) severity of depressive symptoms Beck depression inventory-II (BDI-II) and (b) distress Outcome Questionnaire-45 (OQ-45).

Then, in order to examine whether ER deficits and their associations with outcome would differ between the diagnostic groups, we examined Pearson correlations between ER variables and outcome variables across different diagnostic groups. After reviewing these correlations, we examined the differences between groups in Pearson correlations for each ER variable. Analyses were done using IBM SPSS (version 27; IBM Corp, 2020), and comparison of correlations was done by transforming each correlation to Fisher Z scores and performing a Z test for differences between independent correlations using the formulas in Snedecor and Cochran (1980).

As this study represents a secondary analysis of existing data, we were limited to the number of participants in the original studies. As such, we conducted a retrospective power analysis in the G^*^power program (Cohen, 1992) that shows that with this sample size, we had an 80% statistical power to detect medium to large effect sizes.

## 3. Results

### 3.1. Differences between groups in study variables

Table 1 shows the differences in ER variables between the diagnostic groups and *post hoc* analyses using the Scheffé *post hoc* criterion for significance. The differences in lack of awareness, lack of clarity, non-acceptance, limited access to ER strategies, difficulties controlling impulses, and difficulties engaging in goal-directed behaviors were found to be significant between the diagnostic groups. The *post hoc* analysis indicated that for the following subscales—non-acceptance, limited access to ER strategies, difficulties controlling impulses, and difficulties engaging in goal-directed behaviors—the average scores of controls were significantly lower than in the other two diagnostic groups, and the latter two groups did not differ from one another. For the lack of clarity subscale, a *post hoc* analysis showed that the average score of controls was significantly lower only than that of ED, and the two psychiatric groups did not differ from one another. A different trend was found for the lack of awareness subscale, in which a *post hoc* analysis showed that the average score of SCZ was significantly lower than that of EDs, whereas controls did not differ from either of the other groups.

**Table 1:** Means, SDs, and one-way ANOVA in DERS, and *post hoc* analyses using the Scheffé *post hoc* criterion.

Measure	SCZ	EDs	Controls	*F* (2, 105)	η^2^	*p* values for Scheffé *post hoc* comparisons		
	M (SD)	M (SD)	M (SD)			SCZ vs. EDs	SCZ vs. Controls	EDs vs. Controls
Lack of awareness	14.66 (4.60)	17.14 (3.16)	16.24 (3.62)	3.83^*^	0.07	0.03^*^	0.22	0.61
Lack of clarity	10.97 (4.19)	12.03 (4.87)	8.91 (2.95)	5.42^*^	0.09	0.55	0.11	0.01^**^
Non-acceptance	16.36 (5.74)	17.78 (6.27)	12.15 (4.90)	9.6^**^	0.16	0.57	<0.01^**^	<0.001^**^
Limited access to emotion regulation strategies	24.38 (7.78)	23.08 (6.84)	16.55 (6.06)	13.21^**^	0.20	0.73	<0.001^***^	<0.01^**^
Difficulties controlling impulses	13.91 (6.34)	14.80 (5.07)	10.19 (4.18)	7.73^*^	0.13	0.78	<0.01^*^	<0.01^*^
Difficulties engaging in goal-directed behaviors	16.72 (4.16)	16.95 (4.79)	12.11 (5.18)	11.99^**^	0.19	0.98	<0.001^**^	<0.001^**^

DERS: Difficulties in ER Scale (Gratz and Roemer, 2004).

* *p* < 0.05.

** *p* < 0.01.

*** *p* < 0.001.

Table 2 presents the differences in the outcome variables between the diagnostic groups and *post hoc* analyses using the Scheffé *post hoc* criterion for significance. The differences in OQ and BDI were found to be significant between the diagnostic groups. The *post hoc* analysis indicated that for depressive symptoms, the average score of controls was significantly lower than that of the other two diagnostic groups, whereas for the general symptoms scale the average score of controls was significantly lower only than that of EDs.

**Table 2:** Means, SDs, and one-way ANOVA in outcome variables and *post hoc* analyses using the Scheffé *post hoc* criterion for differences between the diagnostic groups.

Measure	SCZ	EDs	Controls	*F* (2, 105)	η^2^	*p* values for Scheffé *post-hoc* comparisons		
	M (SD)	M (SD)	M (SD)			SCZ vs. EDs	SCZ vs. Controls	EDs vs. Controls
General symptom severity (OQ-45)	74.19 (24.72)	84.26 (19.30)	59.80 (19.79)	11.87^**^	0.18	0.14	0.20	<0.001^***^
Depressive symptoms (BDI-II)	21.86 (12.06)	24.82 (10.73)	12.14 (8.99)	13.92^**^	0.21	0.50	<0.001^***^	<0.001^***^

OQ-45: Outcome Questionnaire-45 (Lambert et al., 1996).

BDI-II; Beck Depression Inventory-II (Beck et al., 1996).

* *p* < 0.05.

** *p* < 0.01.

*** *p* < 0.001.

### 3.2. Relations between ER variables and outcome variables in each of the diagnostic groups

As can be seen in Table 3, for the most part, ER variables were found to be significantly correlated with both outcome variables (i.e., depression and the general symptoms scales in at least one diagnostic group). Of note, only for SCZ were the correlations between all ER variables and outcome variables found to be significant. The associations of the subscale of limited access to ER strategies with both outcome variables were found to be significant across all diagnostic groups. Lack of clarity, difficulties controlling impulses, and non-acceptance were found to be significantly correlated with both outcome variables for SCZ and controls, but not for EDs. Lack of awareness and difficulties engaging in goal-directed behavior were found to be significantly correlated with both outcome variables only for SCZ.

**Table 3:** Pearson correlations between DERS dimensions and outcome, across the diagnostic groups (*n* = 108).

Variables	General symptom severity OQ-45			Depressive symptoms BDI-II		
	SCZ (*n* = 36)	ED (*n* = 36)	Controls (*n* = 36)	SCZ (*n* = 36)	ED (*n* = 36)	Controls (*n* = 36)
Lack of Awareness	0.29^*^	0.03	0.20	0.32^*^	−0.03	0.14
Lack of Clarity	0.41^*^	−0.01	0.38^*^	0.57^**^	0.06	0.44^**^
Non-Acceptance	0.50^**^	0.27	0.51^**^	0.42^*^	0.35^*^	0.59^**^
Limited Access to ER Strategies	0.61^**^	0.58^**^	0.37^*^	0.58^**^	0.61^**^	0.41^*^
Difficulties Controlling Impulses	0.54^**^	0.25	0.69^**^	0.48^**^	0.31	0.74^**^
Difficulties in Goal-Directed Behaviors	0.67^**^	0.31	0.19	0.48^**^	0.39^*^	0.42^**^

* *p* < 0.05.

** *p* < 0.01.

*** *p* < 0.001.

Results from the comparisons of correlations between ER variables and outcome variables from the different diagnostic groups are presented in Table 4. As can be seen, the association between the lack of clarity and difficulties in goal-directed behaviors dimensions with general symptom severity was significantly stronger among SCZ than among EDs. In addition, the association between lack of clarity and depressive symptoms was significantly stronger among SCZ than among EDs. Finally, lack of clarity and difficulties controlling impulses were associated with both outcome measures significantly more among controls than among EDs.

**Table 4:** Significance of differences between the correlations of DERS and outcomes, in the three diagnostic groups.

Variables	General symptom severity OQ-45			Depressive symptoms BDI-II		
	SCZ vs. ED	SCZ vs. Controls	ED vs. Controls	SCZ vs. ED	SCZ vs. Controls	ED vs. Controls
Lack of awareness	0.14	0.36	0.24	0.08	0.22	0.25
Lack of clarity	0.04^*^	0.45	0.05^*^	<0.01^**^	0.24	0.05^*^
Non-acceptance	0.13	0.49	0.13	0.37	0.18	0.10
Limited access to ER strategies	0.45	0.10	0.12	0.43	0.18	0.14
Difficulties controlling impulses	0.08	0.16	<0.01^**^	0.21	0.04^*^	<0.01^**^
Difficulties in goal-directed behaviors	0.02^*^	<0.01^**^	0.47	0.34	0.39	0.44

* *p* < 0.05.

** *p* < 0.01.

## 4. Discussion

In the current study, we examined differences in levels of ER ability difficulties and their associations with psychological outcome across different diagnostic groups. Results of the current study indicate that most ER abilities and outcome measures are more impaired in the clinical groups (i.e., SCZ or EDs) than in controls. In addition, the study emphasizes the central role of ER abilities in psychological outcome beyond specific diagnosis, as it was associated with outcome in all groups. However, the importance of ER abilities as associated with outcome seems to be especially relevant for SCZ as compared with EDs, as all dimensions of ER abilities were found to be positively related to outcome measures of depression and the general symptoms scores in this population, and as two ER dimensions (lack of clarity and difficulties in goal-directed behaviors) had significantly stronger correlations with outcome measures among SCZ than among EDs.

The finding that difficulties in ER abilities were higher in both diagnostic groups (i.e., SCZ and EDs) than in the control group is consistent with previous studies that found that emotional difficulties and ER strategy deficits were higher in clinical populations than controls (Trémeau, 2006; Aldao et al., 2010; Lukas et al., 2018; Lincoln et al., 2022). Furthermore, there were no significant differences between SCZ and EDs in difficulties in ER abilities, in line with previous literature showing similar maladaptive ER strategies and ER skills among SCZ and EDs (Livingstone et al., 2009; Lincoln et al., 2015). This finding may imply that SCZ and EDs share difficulties in relating and responding to emotional distress. Further examination is needed to extend this finding and explore its origins and broader consequences for clinical manifestations. Finally, difficulties in ER abilities were more strongly associated with outcome in the SCZ group than in the EDs group and the control group. All of the dimensions of ER abilities were associated with general symptom severity and depressive symptoms among SCZ. In addition, lack of clarity and difficulties in goal-directed behaviors were significantly more associated with outcome among the SCZ group than among the EDs group. Taken together, it seems that the type and level of difficulties in ER abilities among SCZ do not differ significantly from the type and level among EDs; nevertheless, ER abilities seem to have more robust and stronger associations with symptomatic outcomes among SCZ than among EDs and controls.

The one observed difference in ER between the two clinical groups was found in the lack of awareness subscale: Difficulties in awareness of emotional responses were significantly lower among SCZ then among EDs. Of note, individuals diagnosed with schizophrenia face deficits in broad awareness-related processes such as insight into the disorder, mentalization, and metacognition (Gilleen et al., 2011; Hasson-Ohayon and Lysaker, 2021) that differ from other psychopathologies. Thus, it might be that emotional awareness as assessed in the current study did not capture the unique challenges that SCZ face in emotional awareness. In addition, previous studies on the psychometric properties of the DERS indicated that the awareness subscale showed relatively poor psychometric properties (Bardeen et al., 2012; Osborne et al., 2017; Hallion et al., 2018). Specifically, it was found that the internal consistency was poor and the DERS as a whole was psychometrically stronger when the awareness subscale was excluded. One possible explanation is that the awareness of emotions subscale assesses a different construct that is less essential to ER (Hallion et al., 2018). Therefore, we suggest interpreting this finding with special caution and to examining more complex dimensions of emotional awareness using additional instruments in order to determine the differences in lack of awareness of emotions and its significance among SCZ and Eds.

In general, our results indicate that the associations of difficulties in ER abilities with psychological outcome is partially transdiagnostic, in line with recent accounts of ER as a transdiagnostic mechanism of change across diagnosis (Gratz and Tull, 2010a,b; Berking and Wupperman, 2012; Sloan et al., 2017). Thus, difficulties in ER abilities might impact poor psychological outcomes, or vice versa, for all individuals, beyond any specific psychiatric diagnosis, although a few dimensions of ER were found to be more strongly associated with outcome in the SCZ group than in the EDs group, in the current study. Moreover, various studies have shown that maladaptive ER abilities are not only related to symptoms and distress, but also may predict and impact them (Nolen-Hoeksema, 2000; Calmes and Roberts, 2007; Hong, 2007; Aldao and Nolen-Hoeksema, 2010; Berking et al., 2011; Rusch et al., 2012). As such, by improving ER abilities, symptoms of different psychiatric diagnoses may decrease.

Furthermore, as mentioned above, we found that the associations between maladaptive ER and psychological outcomes were especially significant for SCZ. This finding is supported by previous research that found that deficits in ER strategies among SCZ were related to maintenance and aggravation of positive symptoms (Ludwig et al., 2019; Liu et al., 2020), negative symptoms (van der Meer et al., 2009), and social functioning (Henry et al., 2008; Kimhy et al., 2012). Based on the central role of ER in determining wellness and recovery among SCZ, Bach and Hayes (2002) suggested that therapy that focuses on adaptive ER abilities, instead of on symptom reduction, would reduce these individuals’ re-hospitalizations and improve their well-being. Our findings may suggest that enhancing emotional clarity and engaging in goal-directed behaviors when experiencing negative emotions might be of particular use for therapeutic interventions with SCZ.

A few of the associations between the ER subscales and the different outcomes were not significant for the EDs group. In addition, emotional clarity and impulse control had significantly stronger correlations with outcome measures among controls than among EDs. Previous findings show that ER abilities predict depressive symptoms both cross-sectionally and longitudinally, among EDs (Berking et al., 2014; Gonçalves et al., 2019). Of note, a stronger association between difficulties in ER abilities and symptoms has been found among women than men (Bender et al., 2012; Gonçalves et al., 2019), indicating the significance of taking gender into account. It could be that difficulties in ER were less associated with symptomatic outcome in our sample because it included mostly men. As the current study represents a preliminary and exploratory investigation, more research is needed in order to determine the relation of difficulties in ER with outcome among EDs. Finally, we did not find significant differences between controls and SCZ Group in part of ER abilities, specifically awareness to emotions and lack of emotional clarity. In addition, SCZ did not differ significantly from controls in general symptoms severity. It could be that the fact that our control group was based on treatment seekers affected these results, and further examination is needed to clarify that.

### 4.1. Limitations, implications, and future directions

Along with its contribution to the literature, the current study also had some limitations. First, its cross-sectional design does not allow for the exploration of causality between the different variables. Second, grouping together people with anxiety or affective disorders into one ED group did not allow for an observation of distinct associations between the different disorders and psychological outcomes and deficits in ER. Third, in this study—as opposed to other studies—controls were those who had sought treatment but did not have a psychiatric diagnosis. In other words, using a control group of non-treatment seekers may have yielded a pattern of results different from that yielded in the current study, which included treatment seekers. Additionally, the different patterns of correlations between SCZ, Eds, and controls could be influenced by the setting of our study that included treatment seekers in all three groups. Fourth, our hypotheses were not pre-registered. Fifth, as this is one of the first studies comparing differences between correlations of ER and outcome across groups, we used a permissive approach to the analysis of the differences between independent correlations. Future research should use a more conservative approach and apply statistical corrections to the comparisons of correlations. In addition, as our sample was relatively small and our investigations were preliminary and exploratory, further examinations with more statistical power are needed in order to determine the association of ER abilities with outcome.

Moreover, a few of the ER variables that were examined in this study (e.g., awareness and clarity of emotions) may be considered aspects of emotional processing or emotional functioning rather than of ER *per se*. Furthermore, other aspects that have been found relevant for ER in other studies, such as monitoring of ER implementation across time (Sheppes et al., 2015), were not examined in this study. In addition, in this study we only focused on two psychological outcome variables. Lastly, the use of self-report measures can cause potential reporting biases (i.e., participants may report socially desirable outcomes).

With these limitations in mind, the current study has several implications. First, difficulties in ER abilities seem to be partially transdiagnostic, as they were evident both among EDs and SCZ. It will be beneficial to systematically explore difficulties in ER abilities across additional diagnostic groups. Furthermore, future studies should assess difficulties in ER abilities *via* observational methods, in addition to self-report measures, as it has been shown that the two types of assessment are not highly correlated (Ganellen, 2007; Kivity and Huppert, 2018).

With regard to clinical implications, there seems to be a partial transdiagnostic effect in the associations of ER abilities with depressive symptoms and psychological distress and functioning. Our findings suggest that exploring ER abilities and their associations with other outcome measures longitudinally could be beneficial for detecting the dynamics of formation and maintenance of clinical symptoms across diagnoses. In addition, in line with recent research indicating the importance of ER abilities in mediating treatment outcomes (Slee et al., 2008; Axelrod et al., 2011; Berking et al., 2011; Berking and Wupperman, 2012; Khakpoor et al., 2019; Igra et al., 2022), our findings point to the potential role of ER abilities as a specific change mechanism in psychotherapy. Therapeutic interventions that emphasize ER abilities and strategies have already been developed, and our findings call for an in-depth exploration of the role of session-by-session specific ER abilities in psychological treatments for SCZ, EDs, and other conditions.

### 4.2. Conclusion

In summary, this study highlights the association between difficulties in ER abilities and depressive symptoms and distress. Our results show that individuals with psychiatric disorders report more deficits in ER and higher levels of depression and distress than do individuals without psychiatric diagnoses. Moreover, there were very few differences in the levels of difficulties in ER abilities between SCZ and EDs, suggesting that SCZ and EDs share difficulties in relating and responding to emotional distress. The associations between difficulties in ER abilities and psychological outcome were found to be more robust and stronger among SCZ than among EDs and controls, suggesting the potential contribution of targeting ER abilities in the treatment of schizophrenia.

## Data availability statement

The raw data supporting the conclusions of this article will be made available by the authors, without undue reservation.

## Ethics statement

The studies involving human participants were reviewed and approved by Ethics committee of the Department of Psychology at Bar-Ilan University. The patients/participants provided their written informed consent to participate in this study.

## Author contributions

LI and SS designed and wrote the first draft of the manuscript. YK conducted statistical analyses and contributed to the first draft of the manuscript. DAS and ALR contributed to the second draft of the manuscript, to literature searches and theoretical paradigms. IHO was the supervisor of the study and contributed to study conception and design at all stages. All authors contributed to and have approved the final manuscript.

## Conflict of interest

The authors declare that the research was conducted in the absence of any commercial or financial relationships that could be construed as a potential conflict of interest.

## Publisher’s note

All claims expressed in this article are solely those of the authors and do not necessarily represent those of their affiliated organizations, or those of the publisher, the editors and the reviewers. Any product that may be evaluated in this article, or claim that may be made by its manufacturer, is not guaranteed or endorsed by the publisher.
